# Fibroblast Growth Factor 21 Protects Against Atrial Remodeling via Reducing Oxidative Stress

**DOI:** 10.3389/fcvm.2021.720581

**Published:** 2021-10-11

**Authors:** Miao Chen, Jiawei Zhong, Zhen Wang, Hongfei Xu, Heng Chen, Xingang Sun, Yunlong Lu, Lu Chen, Xudong Xie, Liangrong Zheng

**Affiliations:** ^1^Department of Cardiology and Atrial Fibrillation Center, The First Affiliated Hospital, School of Medicine, Zhejiang University, Hangzhou, China; ^2^Department of Cardiovascular Surgery, The First Affiliated Hospital, School of Medicine, Zhejiang University, Hangzhou, China

**Keywords:** fibroblast growth factor 21, atrial remodeling, atrial fibrillation, oxidative stress, nuclear factor erythroid 2-related factor 2

## Abstract

**Aim:** The structural and electrical changes in the atrium, also known as atrial remodeling, are the main characteristics of atrial fibrillation (AF). Fibroblast growth factor 21 (Fgf21) is an important endocrine factor, which has been shown to play an important role in cardiovascular diseases. However, the effects of Fgf21 on atrial remodeling have not been addressed yet. The purpose of the present study is to evaluate the effects of Fgf21 on atrial remodeling.

**Methods and Results:** Adult mice were treated with Ang II, and randomly administrated with or without Fgf21 for 2 weeks. The susceptibility to AF was assessed by electrical stimulation and optical mapping techniques. Here, we found that Fgf21 administration attenuated the inducibility of atrial fibrillation/atrial tachycardia (AF/AT), improved epicardial conduction velocity in the mice atria. Mechanistically, Fgf21 protected against atrial fibrosis and reduced oxidative stress of the atria. Consistently, *in vitro* study also demonstrated that Fgf21 blocked the upregulation of collagen by Tgf-β in fibroblasts and attenuated tachypacing-induced oxidative stress including reactive oxygen species (ROS), Tgf-β, and ox-CaMKII in atrial myocytes. We further found that Fgf21 attenuated oxidative stress by inducing antioxidant genes, such as SOD2 and UCP3. Fgf21 also improved tachypacing-induced myofibril degradation, downregulation of L-type calcium channel, and upregulation of p-RyR2, which implicated protective effects of Fgf21 on structural and electrical remodeling in the atria. Moreover, Nrf2 was identified as a downstream of Fgf21 and partly mediated Fgf21-induced antioxidant gene expression in atrial myocytes.

**Conclusion:** Fgf21 administration effectively suppressed atrial remodeling by reducing oxidative stress, which provides a novel therapeutic insight for AF.

## Introduction

Atrial fibrillation (AF) is the most common cardiac arrhythmia, which is related to structural and electrical changes in the atria. One major feature of structural remodeling is atrial fibrosis, which leads to a heterogeneity of conduction and reduced conduction velocity. Another feature of structural remodeling is the degradation of myofibrils, also called myolysis. Furthermore, electrical remodeling is caused by changes of ion channels mainly including calcium channels and potassium channels. Although the mechanism underlying the atrial remodeling is not fully understood, an increasing number of studies suggested that oxidative stress is markedly related to the process of the atrial remodeling (1, 2).

Fibroblast growth factor 21 (Fgf21) is a member of the FGF family, which acts as an endocrine factor. Fgf21 is mainly produced and secreted by the liver, and it also can be produced by other organs, including the skeletal muscle, the adipose tissue, and the heart (3). Fgf21 can be produced by various pathological conditions, such as oxidative stress and pro-inflammatory condition, to provide a negative-feedback protective effect (4, 5). The association between Fgf21 and some cardiovascular diseases, such as myocardial infarction, hypertensive heart disease, and cardiac hypertrophy has been intensely investigated (6–8). With consideration of AF, clinical evidence revealed that Fgf21 levels increased in plasma and atrial tissues in patients with AF (9). However, the function of Fgf21 in the atrial remodeling of AF remains unaddressed.

In light of the potential association between Fgf21 and AF, we aim to investigate the preventive effect of Fgf21 against atrial remodeling in the present study.

## Materials and Methods

### Human Sample and Immunohistochemical Stain

Eleven (11 patients with SR and 8 patients with AF) surgical specimens of atrial tissues were obtained from patients admitted to the First Affiliated Hospital of Zhejiang University School of Medicine. The obtained atrial samples were fixed with 4% paraformaldehyde. Paraffin-embedded atrial tissues were sliced at 5-μm thickness by Servicebio (Wuhan City, China). After deparaffinization, rehydration, and antigen retrieval, Fgf21 primary antibody (1:200) was applied. Then the avidin–biotin–peroxidase complex method was performed by using diaminobenzidine as a substrate and hematoxylin as a counterstain. Two slices were taken from each sample, and three random visual fields were selected. The integrated optical density (IOD) value, which reflects a positively stained area, was measured using Image J software. The clinical characteristics of patients are presented in Supplementary Table 1.

### Mouse Models

C57/BL6 male mice aged 6–8 weeks were purchased from the Zhejiang Academy of Medical Science (China). The average body weight of these mice was 20–25 g. The mice were randomly divided into three groups: control, AngII+saline, and AngII+Fgf21; each group contained six mice. Osmotic mini pumps containing AngII (1 mg/kg/day) were implanted subcutaneously for 2 weeks. AngII-treated mice were randomized to receive Fgf21 (2.5 μg/kg/d) or saline by intraperitoneally (i.p.) injecting for 2 weeks. The studies of animals were approved by the Institution Animal Experimental Ethics Committee of the First Affiliated Hospital of Zhejiang University (Reference Number: 20201554). All animal experiments were conducted in accordance with the approved guidelines of the animal ethics committee of the hospital.

### Electrical and Optical Mapping of *ex vivo* Heart Preparations

To study the AF inducibility and electrical conduction patterns of atria, we conducted high resolution of electrical and optical mapping by using methods, which have been described previously (10). Briefly, the heart was removed and mounted onto a Langendorff perfusion system immediately. Extracellular potentials (ECP) of the atrium were recorded by using a multi-electrode array mapping system (EMS64-USB-1003, MappingLab Ltd., UK). Two multi-electrode arrays (MEA) with 64 electrodes were employed for right and left atrial simultaneous recordings. The intra-electrode distance of MEA was 0.2857 mm and the overall area was 3.9996 mm^2^. ECP signals were recorded using EMapRecord 5.0 software (MappingLab Ltd., UK) and stored in the computer for subsequent analysis using EMapScope 5.0 and OMapScope5.7 software (MappingLab Ltd., UK).

A stimulator was placed on the right atrium to assess AF inducibility by using burst pacing. The protocol of burst pacing was as follows: 50 Hz burst pacing was applied for 10 s and then stabilized for 4 min. The burst pacing was repeated eight times with 4 min of stabilization each time. AF was defined as a rapid and irregular atrial rhythm (rapid and irregular ECP) lasting at least 1 s, and atrial tachycardia (AT) was defined as a rapid and regular atrial rhythm (rapid and regular ECP) lasting at least 1 s.

Then the optical mapping for both atria was performed, we used excitation–contraction uncoupler Blebbistatin (10 μM) to arrest cardiac motion. After stabilization, the membrane potential fluorescent dye Rh237 (dose, 1 mg/ml, 40 μl) was loaded to the perfusate to optically map membrane potential. The hearts were illuminated with two 530 nm LEDs (LEDC-2001, MappingLab Ltd.) that their emissions were bandpass filtered (wavelengths 530 ± 20 nm). The fluorescence signals were passed through a 550-nm long-pass filter and were divided by a dichroic mirror (cut off = 638 nm). Fluorescence light with wavelengths above 638 nm was passed through a 700 nm long-pass filter and then recorded by the camera for voltage signals. Fluorescence light below 638 nm was passed through a bandpass filter (585 ± 40 nm) then recorded by the camera for calcium signals (OMS-PCIE-2002, MappingLab Ltd). The changes of fluorescence were captured by a camera at 900 frames/s and spatial resolution was 128 by 128 pixels. The recorded signals were analyzed by using EMapScope 5.0 and OMapScope5.7 software (MappingLab Ltd., UK).

### HL-1 Atrial Myocytes Culture and Pacing

HL-1 cells were purchased from Sigma-Aldrich (SCC065). HL-1 cells were cultured with Calycomb Medium (Sigma, United States) supplemented with 10% fetal bovine serum (FBS), 1% Penicillin/Streptomycin, 2 mM L-Glutamine, and 0.1 mM Norepinephrine. The cells were cultured in flasks coated with 5 μg/ml fibronectin and 0.02% gelatin at 37°C in a 5% CO_2_ atmosphere. Then the cells were cultured in six-well dishes to induce tachypacing using a cell pacing system (Ionoptix, USA). The cells were subjected to rapid field stimulation for 24 h at 7 Hz (20 V, 5 ms). In a few experiments, HL-1 cells were pre-treated with 20 nM Fgf21 for 24 h before rapid field stimulation.

### Primary Cardiomyocytes and Atrial Fibroblasts Culture

Primary murine cardiomyocytes and cardiac fibroblasts (CFs) obtained from 1- to 2-day neonatal mice were isolated according to previous protocol (11). In short, cardiomyocytes and fibroblasts were collected by performing differential adhesion for 1.5 h according to different wall-adherence durations of fibroblasts and cardiomyocytes. The isolated cardiomyocytes and fibroblasts were cultured in six-well culture dishes in DMEM with 10% FBS. As reported previously (12), rat cardiac fibroblasts cultured on a rigid substrate show progressive fibroblast-to-myofibroblast differentiation, but passage 0 (P0) and passage 1 (P1) were still considered as cardiac fibroblasts. P0 cells were used in our experiment and stimulated by Tgf-β1 (with or without 20 nM Fgf21) in 24 h after adhering.

### Immunofluorescence Analysis

The expression of myosin heavy chain (MHC) was measured by using immunofluorescence analysis. First, the cells were washed three times with a phosphate buffered saline. After fixing with 4% paraformaldehyde, the cell membranes were permeabilized with 0.1% Triton X-100 for 10 min at RT. Then the cells were covered with 10% donkey serum to block for 30 min, and incubated with primary antibody (diluted with PBS appropriately) overnight at 4°C. Next, cover cells with secondary antibody (FITC-conjugated) and nuclei were visualized by staining with DAPI. The immunofluorescence staining of the mouse atrial tissue was similar to cells. After deparaffinization, rehydration, and serum blocking, the slides were incubated with a primary antibody against ox-CaMKII (GeneTex). Under a confocal laser scanning microscope (Leica, Germany), three random visual fields were selected and analyzed.

### Real-Time Quantitative PCR

Total RNA was extracted from HL-1 cells with Trizol reagent according to the protocol provided by the manufacturer. Then the PrimeScript™ RT Master Mix (Takara, Japan) was used to synthesize cDNA. Real-time quantitative RT-PCR reactions were performed using 1 μl of diluted cDNA. The primers used in this study are listed as follows:

FGF21: Forward-5′CTGCTGGGGGTCTACCAAG3′, Reverse-5′CTGCGCCTACCACTGTTCC3′; FGFR1: Forward-5′GGAACAGCAGGAGAAGAC3′, Reverse-5′GAGAGGAATATGAGGAGTAGG3′; FGFR3: Forward-5′CCAGGTTCATAGCGTTTAG3′, Reverse-5′TAGGAGAGCCATTCAAGC3′; UCP3: Forward-5′AGCAGCAGGACTCAGAATC3′, Reverse-5′GAGCAGGAGGAAGTGTGG3′; SOD2: Forward-5′GAAATCCGCCTGCCTCTG3′, Reverse-5′AATGCCTTCTTCTGGTGTGG3′; Prdx5: Forward-5′TGATAGACAACGGCATAGTG3′, Reverse-5′GAGATGGGAGAGTCAGAGG3′; CAT: Forward-5′TGCGGACATTCTACACAAAG3′, Reverse-5′ATTGCGTTCTTAGGCTTCTC3′; GPX1: Forward-5′GCATTGGCTTGGTGATTACTG′, Reverse-5′TCATTAGGTGGAAAGGCATCG3′; Sqstm1: Forward-5′CACAGGCACAGAAGACAAG3′, Reverse-5′ACCGACTCCAAGGCTATC3′.

### Detection of Reactive Oxygen Species

For intracellular oxidative stress, fluorescent dye dihydroethidium (DHE) was used to measure reactive oxygen species (ROS) and detected by confocal microscope (Olympus, Japan). After rapidly pacing, HL-1 cells were incubated with DHE (10 μmol/L) for 30 min at 37°C in the dark. DHE was excited by an argon laser at 488 nm. The protocol for detecting the ROS levels of mouse atrial tissue was similar to cells by using frozen slides. Under a fluorescence microscopy, three random visual fields were selected and analyzed.

### Western Blot

Total proteins were isolated from cultured cells by using a lysis buffer. Then equal amounts of protein lysates were separated by SDS-page gel electrophoresis. After transferring onto a PVDF membrane, protein was incubated with primary antibodies against Fgf21, Sirt1, Troponin I, t-CamKII, Calpian 1, NOX2, NOX4, GAPDH (Abcam), Nrf2, MHC, SOD2, UCP3, p-RyR2, β-Klotho (Abclonal), ox-CamKII (GeneTex), RyR2, FgfR1 (Proteintech), L-type calcium channel α1C-subunit (LCC) (Affinity), α-SMA, Collagen-1A1, CTGF, and Tubulin (Servicebio). Then they were incubated with a secondary antibody conjugated to HRP (1:5,000) for 1 h. Signals of proteins were identified by chemiluminescence and quantified by densitometry.

### siRNA Transfection

siRNA transfection was performed using Lipofectamine 3000 (Invitrogen) when HL-1 cells reach 80% confluence. Fifty nM of control siRNA, Sirt1 siRNA, or Nrf2 siRNA duplexes were incubated with 3.75 μl siRNA transfection reagent for 15 min at room temperature. Then these mixtures were added to HL-1 cells. At 48 h after transfection with siRNA, the cells were treated with Fgf21 for 24 h and then were stimulated with rapid electrical field.

### Statistical Analysis

Continuous data was presented as the mean ± SEM and compared by unpaired Student's *t*-test or one-way ANOVA. Categorical data was given as numbers and percentages and compared by Fisher's exact text. All data analysis was performed with SPSS 21.0 (SPSS, Inc., Chicago, IL, USA). The *p*-values were two-sided and a *p* < 0.05 was considered statistically significant.

## Results

### Elevated Fibroblast Growth Factor 21 Expression Was Associated With Atrial Remodeling

**Figure 1:**
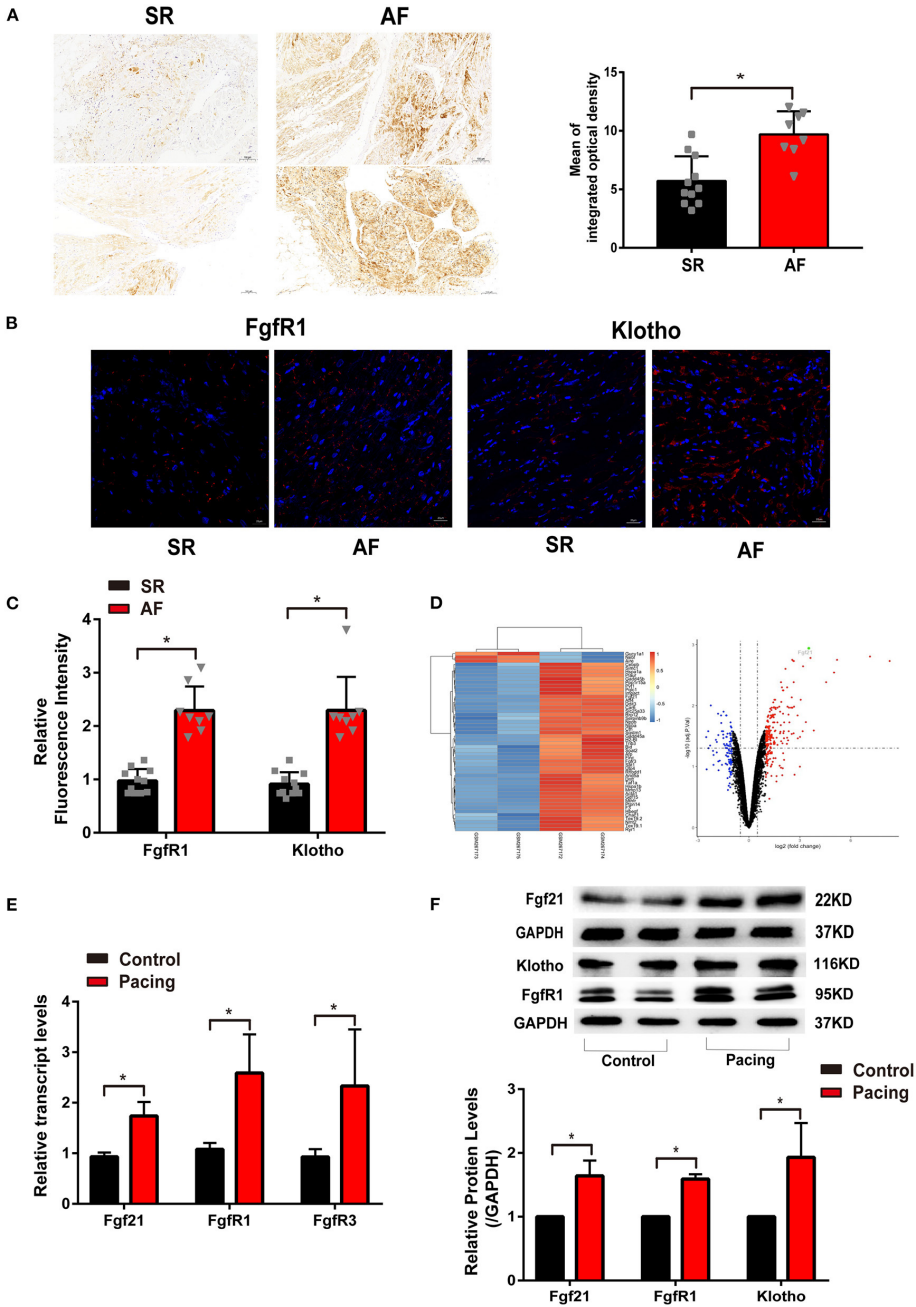
The level of fibroblast growth factor 21 (Fgf21) increased in the atrial tissue of patients with atrial fibrillation (AF) and cell models of atrial remodeling. **(A)** Representative immunohistochemistry image of Fgf21 in sarcoplastic reticulum (SR) and AF patients. Three sections per tissue sample were prepared, and three random pictures were taken from each section. IOD, integrated optical density. **(B)** Representative immunofluorescence images of FgfR1 and β-Klotho in SR and AF patients. **(C)** Summary of the relative fluorescence intensity of FgfR1 and β-Klotho in the atrial tissue of SR and AF patients. **(D)** One clustering diagram and one volcano plot of the differentially expressed genes (DEG) screening. Volcano plot for the expression change of genes, in which the abscissa represents the fold change (log2) and the ordinate represents the adj. *p*-value (–log10). The black spots represent unchanged genes between pacing and non-pacing samples; the red spots represent upregulated DEGs; the blue spots represent downregulated DEGs. **(E)** RT-PCR analysis for the mRNA expression levels of Fgf21, FgfR1, and FgfR3 in tachypaced HL-1 cells. **(F)** The expression levels of Fgf2, FgfR1, and β-Klotho in tachypaced HL-1 cells were assessed by Western Blot. The expression levels of GAPDH were used as an internal control. *p < 0.05.

To investigate the relationship between Fgf21 and atrial remodeling, we collected atrial tissue samples from 11 SR patients and eight AF patients who underwent cardiac surgery. The characteristics of the patients were recorded (Supplemental Table 1). Immunohistochemistry was conducted in each tissue section to evaluate the expression levels of Fgf21. The results showed that the distribution of Fgf21 in atrial tissues of AF patients was higher than that of SR patients (Figure 1A). Given that Fgf21 exert function mainly through fibroblast growth factor 21 receptor-1(FgfR1) and its co-receptor β-Klotho in the heart, we also performed immunofluorescence to assess expression level of FgfR1 and β-Klotho in SR and AF patients. The results revealed that the expression of FgfR1 and its co-receptor β-Klotho increased in the atrial tissue of AF patients (Figures 1B,C).

Previous studies have illustrated that rapid stimulation of HL-1 cells resembles the phenotypic characteristics of atrial remodeling induced by tachycardia *in vivo* (2, 13, 14). First, we analyzed related array data from the GEO database (GSE10598) (15). The results of the dataset revealed that rapidly stimulating HL-1 cells for 24 h increased Fgf21 expression (Figure 1D). To validate the effect of tachypacing on Fgf21 expression, we used this atrial-derived model. As previously described, the rapid stimulation rate was 4–10 Hz, so we used 7 Hz in this study (14, 16). Our results also showed that the mRNA level of Fgf21, FgfR1, as well as FgfR3 increased in HL-1 cells (Figure 1E). Additionally, the result of Western blot also showed that the protein level of Fgf21, FgfR1, and β-Klotho increased in HL-1 after tachypacing (Figure 1F). These findings imply that the activation of atrial myocytes upregulates Fgf21 and its receptor expression level.

### Fibroblast Growth Factor 21 Reduces Atrial Fibrillation/Atrial Tachycardia Inducibility in Angiotensin II-Treated Mice

**Figure 2:**
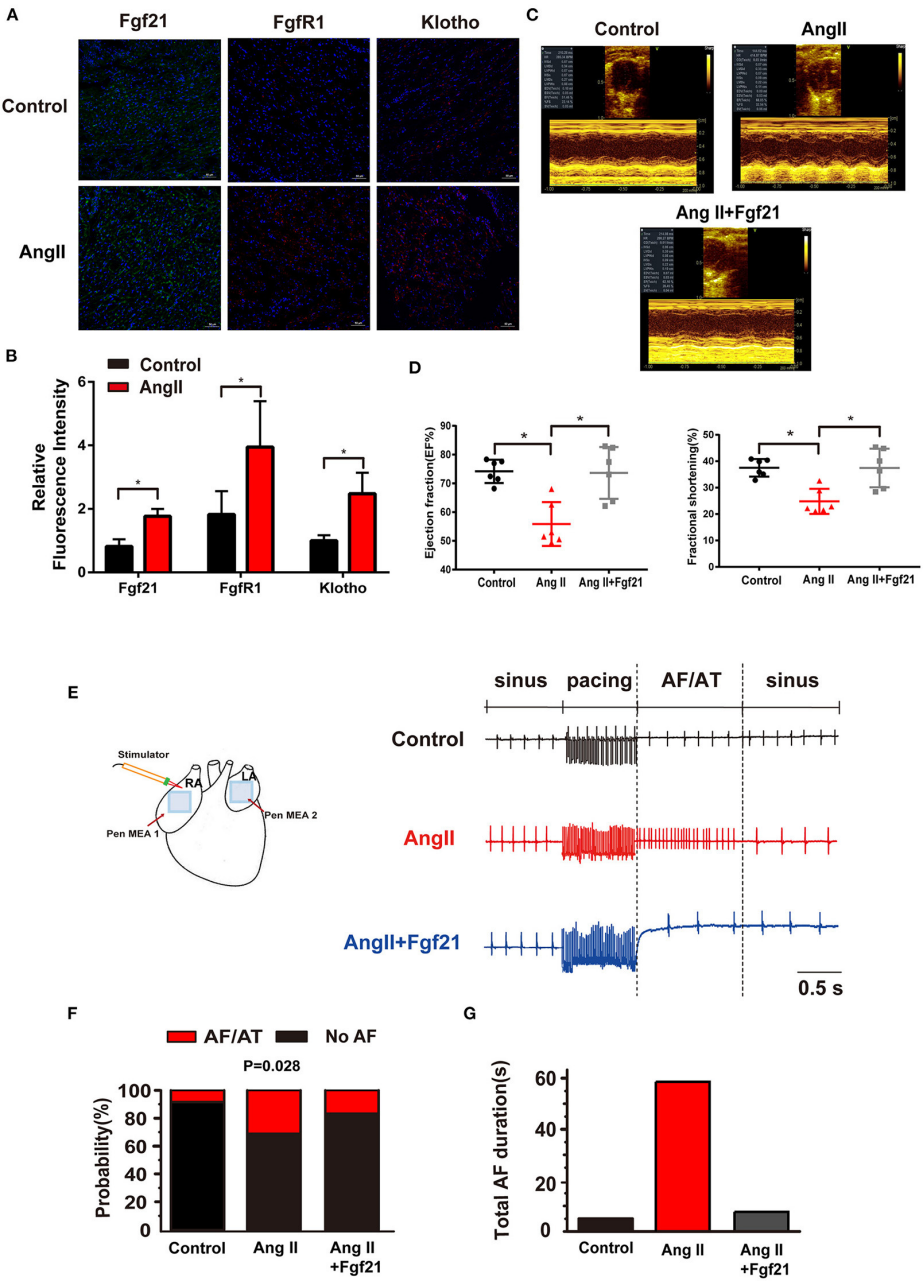
Echocardiography and atrial electrophysiology in Ang II mice with or without treatment of Fgf21. **(A)** Representative immunofluorescence images of Fgf21, FgfR1, and β-Klotho in control and AngII-treated mice. **(B)** Summary of the relative fluorescence intensity of Fgf21, FgfR1, and β-Klotho in the atrial tissue of control and AngII-treated mice. **(C)** Representative M-mode images of each group: control, AngII, and AngII+Fgf21. **(D)** Summary of the effects of Fgf21 on ejection fraction and fractional shortening. **(E)** A schematic illustration of the experimental manipulation of electrical mapping. Representative extracellular potentials (ECP) of the atrium illustrate the induction of AF/AT in each group mouse following burst pacing in the right atrium. **(F)** Summary of inducibility of AF/AT in each group mouse. **(G)** Summary of total duration time of AF/AT in each group mouse that were induced into AF/AT. *p < 0.05, *n* = 6 mice per group.

Previous studies showed that AF was more prevalent in hypertension and elevated angiotensin II (10). Ang II can cause electrical and structural remodeling in atria, which increases susceptibility to AF. Here, we investigated the effects of Fgf21 on atrial remodeling in mice treated with Ang II. We observed the expression level of Fgf21, FgfR1, and β-Klotho increased in the atria of AngII-treated mice, which was similar to the finding of AF patients' atrial tissue (Figures 2A,B). First of all, cardiac function markedly improved in Fgf21-treated AngII mice compared with NS-treated AngII mice (Figures 2C,D). We further observed the effect of Fgf21 on the susceptibility to AngII by an electrical stimulation to induce AF/AT in mice. As shown in Figure 2E, two MEA were respectively placed on the surface of the LA and RA to record extracellular potentials, and a stimulator was placed on the RA to induce AF by using burst pacing. Figure 2E represented typical recordings of AF/AT, and our results showed that the vulnerability to electrical-induced AF/AT was markedly increased in AngII mice, which was attenuated by Fgf21 treatment (from 28.5% to 14.6%) (Figure 2F). Additionally, the duration of total AF/AT episodes (sum of AF/AT duration times) was shorter in Fgf21-treated AngII mice as compared with NS-treated AngII mice (Figure 2G). These findings suggested that Fgf21 exerts anti-arrhythmic effects.

### Effects of Fibroblast Growth Factor 21 on Electrical Conduction in the Atria

**Figure 3:**
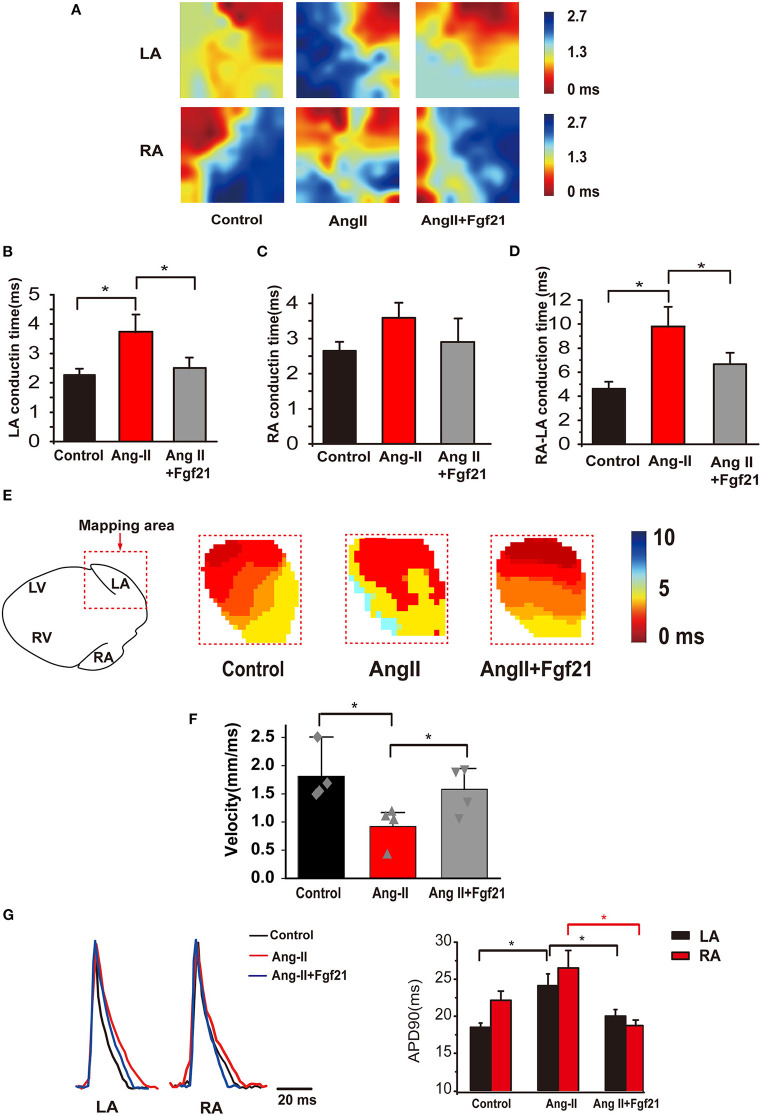
Atrial electrical conduction properties in Ang II mice with or without treatment of Fgf21. **(A)** Representative activation maps from electrical mapping in isolated atrial preparations from each group of mice. Red color indicates the earliest activation time and the blue color indicates the latest activation time. **(B,C)** Summary of the right and left atrial conduction time in sinus rhythm. **(D)** Summary of the conduction time of the right atria to the left atria in sinus rhythm. **(E)** Representative activation maps of the left atria from optical mapping from each group of mice. Red color indicates the earliest activation time and the blue color indicates the latest activation time. **(F)** Summary of the conduction velocity of the left atria in sinus rhythm. **(G)** Representative traces of optical action potentials by optical mapping in atria from each group mouse. Summary of the mean APD90 of mice hearts from each group. *p < 0.05, *n* = 6 mice per group.

Aberrant cardiac electrical conduction is an important characteristic of atrial arrhythmias in AngII-treated mice. We, thus, characterized the effects of Fgf21 treatment on cardiac epicardial conduction. Representative activation maps from electrical mapping illustrated that conduction time of the left atria was significantly prolonged in AngII-treated hearts, while the conduction time of the right atria was not significantly changed (Figures 3A–C). Nonetheless, prolonged atrial conduction time was markedly attenuated by Fgf21 administration (Figure 3B). Fgf21 also attenuated the prolongation of conduction time from the right atria to the left atria (Figure 3D). Because conduction time cannot be entirely equivalent to conductive velocity, we performed optical mapping to further evaluate electrical conduction. The representative activation maps of optical mapping for the left atria are shown in Figure 3E. The results from optical mapping were consistent with the findings of electrical mapping, which revealed the reduced conduction velocity was recovered by Fgf21 administration (Figure 3F). Additionally, the results from optical mapping revealed that the APD90 was significantly prolonged in both the left and right atria of AngII mice, while Fgf21 administration attenuated these changes (Figure 3G). These results revealed that Fgf21 improves the cardiac electrical conduction of AngII mice atria.

### Fibroblast Growth Factor 21 Protects Against Cardiac Fibrosis

**Figure 4:**
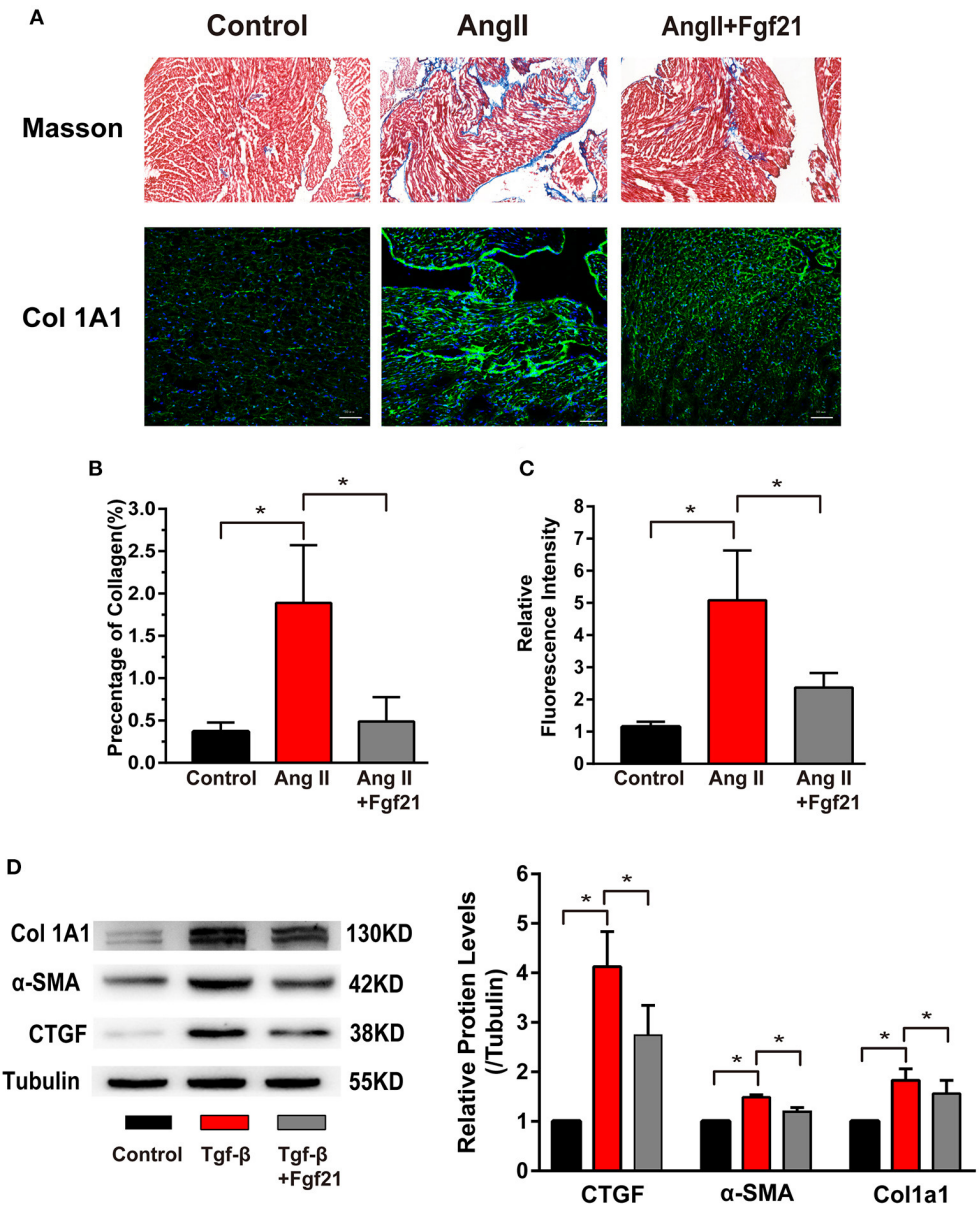
Effects of Fgf21 on atrial fibrosis. **(A)** Representative photomicrographs of atria slices from each group mouse stained with Masson and immunohistochemistry of collagen I. Scale bar indicates 100/50 μm. **(B)** Summary of the total collagen volume in the atria of each group mouse. **(C)** Summary of the relative fluorescence intensity of collagen I in the atria of each group mouse. **(D)** The expression of Col 1A1, α-SMA, and CTGF at protein levels in each group of fibroblasts. *p < 0.05, *n* = 6 mice per group.

Atrial fibrosis is a prominent characteristic of structural remodeling, which leads to altered conduction. Then we evaluated whether Fgf21 has a protective effect on cardiac fibrosis. Mice treated with AngII displayed elevated atrial fibrosis by Masson stain and collagen I immunostaining. Fgf21 treatment markedly improved the degree of atrial fibrosis (Figures 4A–C). *In vitro*, we used rat cardiac fibroblast to evaluate the protective effect of Fgf21 on cardiac fibrosis. We confirmed that the protein levels of collagen 1A1, α-SMA, and CTGF were increased in fibroblasts activated by TGF-β, and Fgf21 attenuated these fibrogenic phenotypes (Figure 4D). These data supported that Fgf21 has a beneficial effect on cardiac fibrosis.

### Fibroblast Growth Factor 21 Reduced Oxidative Stress in Cardiomyocytes

**Figure 5:**
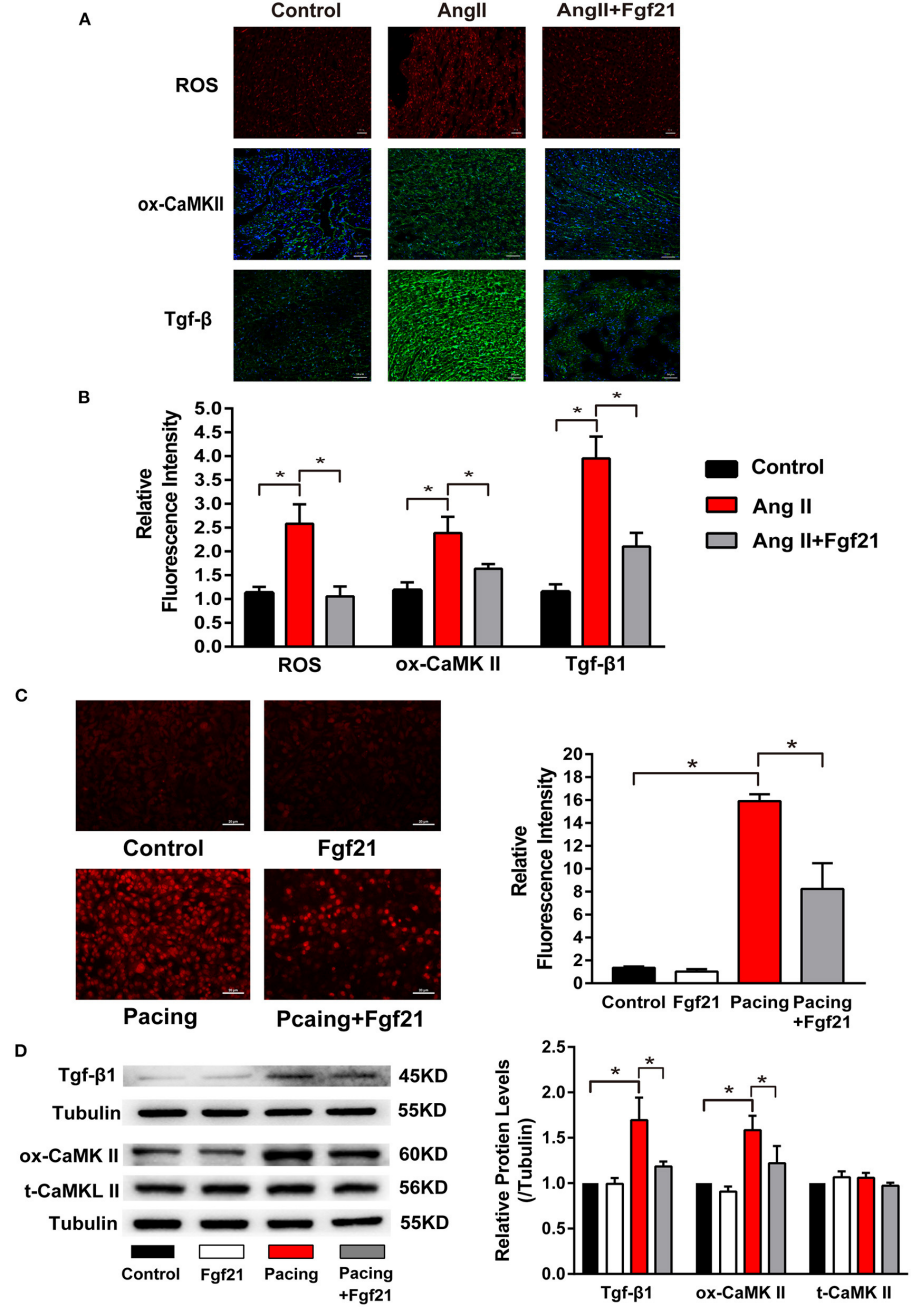
The effects of Fgf21 on oxidative stress in the atria. **(A)** Representative photomicrographs of the atria slices from each group mouse stained with immunohistochemistry of reactive oxygen species (ROS), ox-CaMKII, and Tgf-β. Scale bar indicates 50 μm. **(B)** Summary of the relative fluorescence intensity of ROS, ox-CaMKII, and Tgf-β in the atria of each group mouse. **(C)** Representative images of ROS detected by dihydroethidium (DHE) and a summary of the relative fluorescence intensity of ROS in each group of HL-1 atrial cells. **(D)** The expression of Tgf-β, ox-CaMKII, and t-CaMKII at protein levels in each group of HL-1 atrial cells. *p < 0.05.

Oxidative stress has been implicated in the structural and electrical remodeling of the atria. We further investigated the effects of Fgf21 on oxidative stress in the atria by ROS immunofluorescence staining. The results showed that the mice treated with AngII caused enhanced oxidative stress, while it was significantly suppressed by Fgf21 administration (Figures 5A,B). Previous studies have demonstrated that increased ROS could lead to elevated levels of ox-CamKII and Tgf-β in the atrium (2, 17). Immunofluorescence staining of the atria illustrated that the levels of ox-CamKII and Tgf-β increased in AngII-treated mice. Nevertheless, increased levels were markedly attenuated by Fgf21 treatment (Figures 5A,B). Similar to *in vivo* experiments, tachypacing enhanced oxidative stress, including ROS production, Tgf-β, and ox-CaMKII in the cellular model of atrial remodeling. The promoting effects of tachypacing on oxidative stress could be blocked by pretreatment of 20 nM Fgf21 in HL-1 myocytes (Figures 5C,D). Additionally, we used primary cardiomyocytes to further clarify the role of Fgf21 in oxidative stress. Treatment of primary cardiomyocytes with Fgf21 alleviated tachypacing-triggered ROS production, including ox-CaMKII and Tgf-β (Supplementary Figures 1A,B).

**Figure 6:**
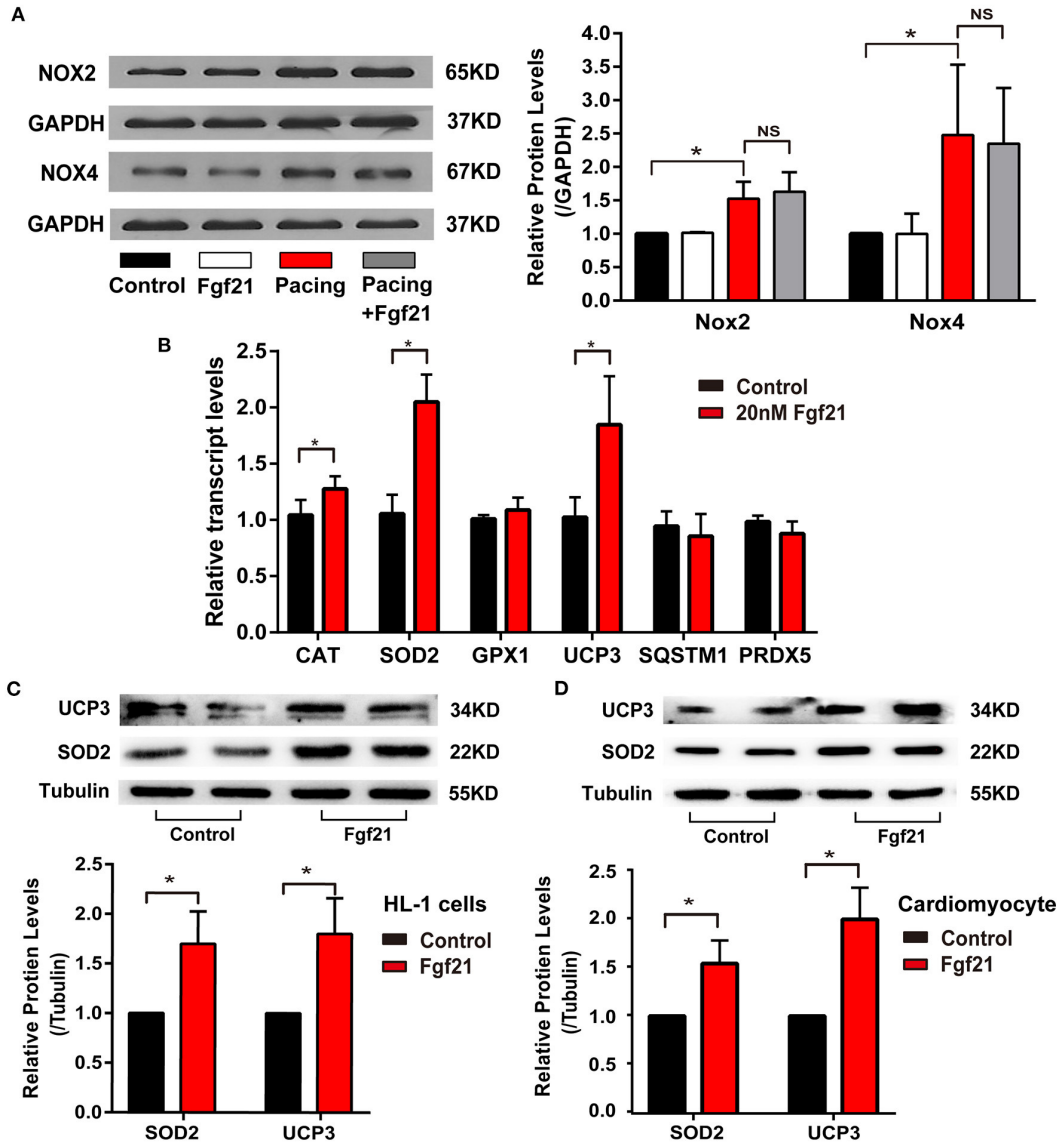
Fgf21 induces anti-oxidative gene expression. **(A)** The expression of Nox2 and Nox4 at protein levels in each group of HL-1 atrial cells. **(B)** The expression of various anti-oxidative genes at mRNA levels in HL-1 after being treated with 20 nM Fgf21. **(C)** The expression of SOD2 and UCP3 at protein levels in HL-1 cells after being treated with 20 nM Fgf21. **(D)** The expression of SOD2 and UCP3 at protein levels in primary cardiomyocytes after being treated with 20 nM Fgf21. *p < 0.05; ns, none sense.

Furthermore, we explored the molecular mechanisms of Fgf21 against oxidative stress. NOX2/4 were the main isoforms of NADPH oxidases in the heart, which is involved in tachycardia-induced oxidative stress (1). However, pre-treated 20 nM Fgf21 for 24 h did not attenuate increased NOX2/4 levels induced by tachypacing (Figure 6A). Therefore, we considered if Fgf21 directly influenced the expression of antioxidant genes in cardiac cells. As shown in Figure 6B,C, treated with 20 nM Fgf21 for 24 h significantly induced the expression of SOD2 and UCP3 in both mRNA and protein levels, and slightly increased the CAT mRNA levels, but the GPX1, SQSTM1, and PRDX5 mRNA levels did not change. The increased levels of SOD2 and UCP3 induced by Fgf21 were also confirmed in primary cardiomyocyte (Figure 6D). These data indicate that Fgf21 directly induced anti-oxidative gene expression against oxidative stress.

### The Effect of Fibroblast Growth Factor 21 on Oxidative Stress-Related Atrial Remodeling

**Figure 7:**
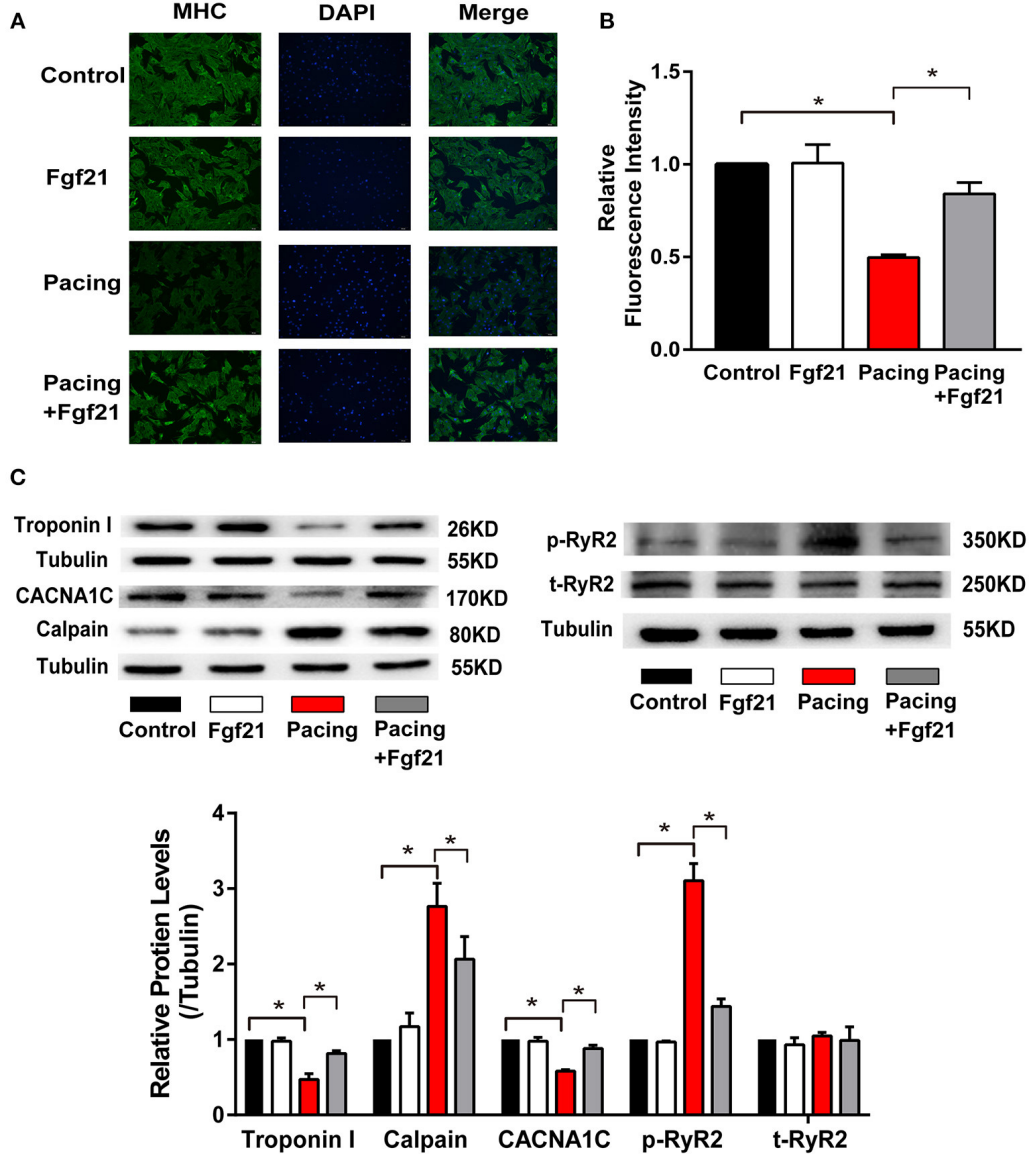
Fgf21 protected against structural and electrical remodeling in the atria. **(A)** Immunofluorescence analysis was performed to analyze myosin heavy chain (MHC). **(B)** Summary of the relative fluorescence intensity of MHC in each group of HL-1 atrial cells. **(C)** The expression of Troponin I, CACNA1C, Calpain, p-RyR2, and t-RyR2 at protein levels in each group of HL-1 atrial cells. *p < 0.05.

Oxidative stress was reported to play a critical role in tachycardia-induced structural and electrical remodeling (2, 14). The above data suggested that Fgf21 could decrease oxidative stress by inducing anti-oxidative gene expression. We further investigated the effects of Fgf21 on atrial remodeling in this cellular model. Myofibril degradation, which was reflected by the degradation of myosin (MHC) and cTroponin I, was a characteristic of structural remodeling. Nevertheless, tachycardia-induced myofibril degradation could be prevented by Fgf21 pre-treatment (Figures 7A,B). Calpain, a Ca^2+^-dependent protease, is reported to be associated with myofibril degradation (18). Furthermore, Fgf21 attenuated tachypacing-induced calpain in protein levels (Figure 7C). These findings in HL-1 cells were also observed in primary cardiomyocytes (Supplementary Figures 1B,C).

It is reported that rapid stimulation of atrial myocytes may contribute to decreased L-type calcium channel (LCC) α1C-subunit expression, which plays an essential role in the process of AF (19). The ryanodine receptor (RyR) is the Ca^2+^ release channel in cardiac myocytes, which is located in the endoplasmic/sarcoplasmic reticulum (SR). Previous studies showed that RyR serine 2814 phosphorylation promotes diastole Ca^2+^ release from SR, which may trigger atrial and ventricular arrhythmia (17). Therefore, we evaluated the protective effect of Fgf21 on electrophysiologic changes. Our results showed that pre-treatment of Fgf21 attenuated tachypacing-induced downregulation of LCC expression and upregulation of p-RyR2 expression (Figure 7C). The above data suggested that Fgf21 protects against atrial remodeling by reducing oxidative stress.

### Fibroblast Growth Factor 21 Induce Anti-Oxidative Genes Expression *via* Nrf2

**Figure 8:**
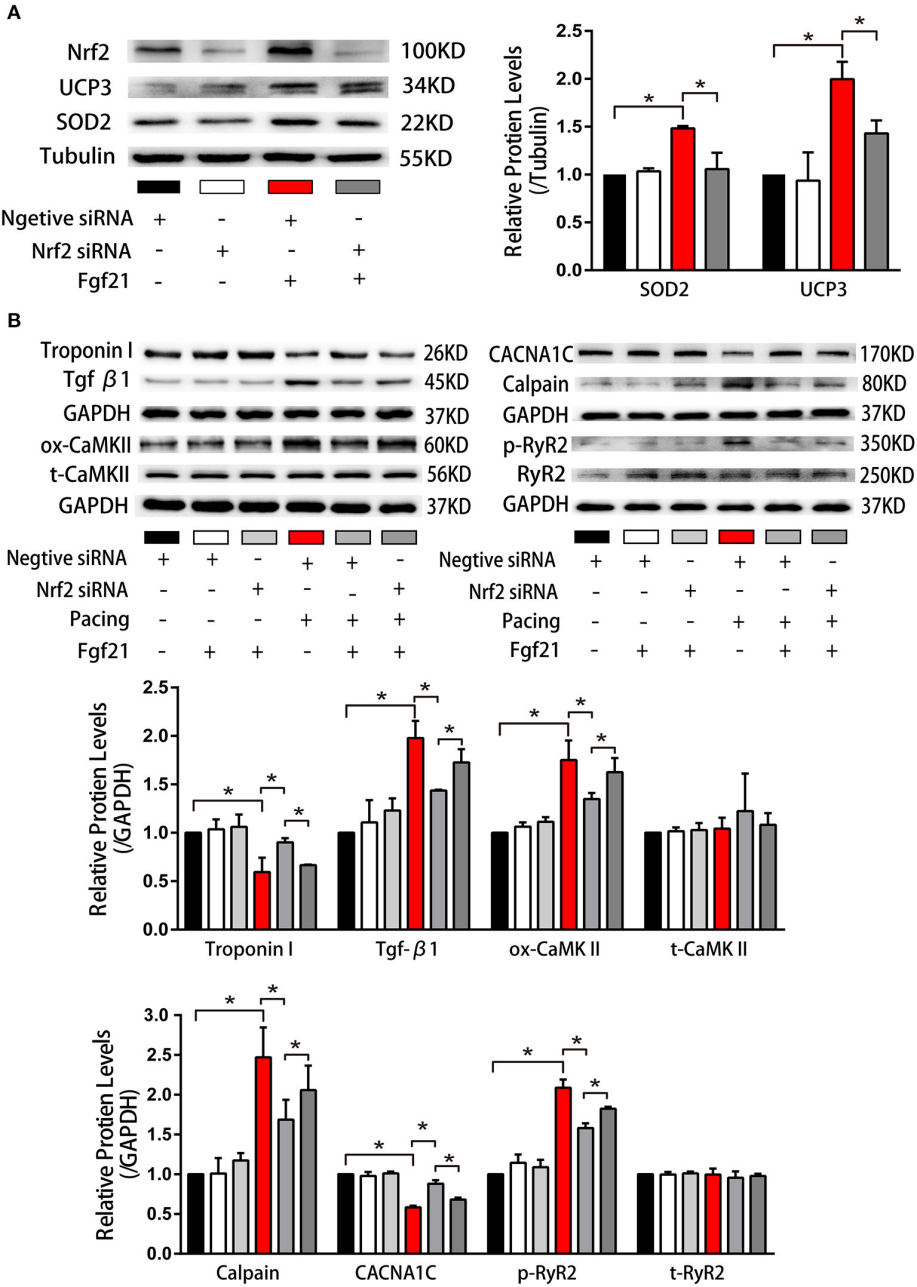
Nuclear factor erythroid 2-related factor 2 (Nrf2) positively regulated anti-oxidative genes expression induced by Fgf21. **(A)** Western blot was conducted to evaluate the expression of SOD2 and UCP3 in HL-1 atrial cells by knockdown of Nrf2 with si-Nrf2. **(B)** Western blot was conducted to evaluate the expression of Troponin I, Tgf-β, ox-CaMKII, t-CaMKII, CACNA1C, Calpain, p-RyR2, and t-RyR2 in HL-1 atrial cells by knockdown of Nrf2 with si-Nrf2. *p < 0.05.

Nuclear factor erythroid 2-related factor 2 (Nrf2) is a critical transcription factor that induces antioxidant gene expression. To further clarify if the upregulation of antioxidant genes (SOD2 and UCP3) in Fgf21-treated myocytes is due to the activation of Nrf2, we have transfected small interfering RNA to inhibit Nrf2 expression. As depicted in Figure 8A, the levels of SOD2 and UCP3 reduced after suppressing Nrf2 expression. Moreover, the beneficial effects of Fgf21 on structural and electrical remodeling in HL-1 cells were abolished by suppressing Nrf2 expression (Figure 8B). These results suggested that Fgf21 plays a cardioprotective role in the suppression of oxidative stress via the regulation of Nrf2.

## Discussion

Atrial remodeling, which is defined as alternations in structural, contractile, and electrical properties, is an essential process in the development of AF. These changes may not only promote the occurrence of triggers of AF, but also promote the formation of a substrate for perpetuation of AF. Oxidative stress is believed to drive atrial remodeling, which contributes to the incidence and development of AF; therefore, antioxidants may become a potential therapy to prevent AF occurrence (20). The present study demonstrated that Fgf21 significantly increased in the atrial tissue of AF patients. Giving exogenous Fgf21 to AngII-treated mice decreased AF/AT inducibility and improved conduction velocity of the atria. Besides, our research suggested that Fgf21 acts as an inducible endogenous factor to prevent oxidative damage and cardiac fibrosis, thus could protect against atrial remodeling. A schematic summary of our findings is shown in Supplementary Figure 2.

Fgf21 is a secreted protein which could be produced by cardiomyocytes exposed to some stress, such as oxidative stress and pro-inflammatory condition. Previous studies suggested that Fgf21 also act as a negative-feedback protective factor that can provide a protective effect in some pathological conditions, including hypertension and alcoholic cardiomyopathy (4, 5). We found that markedly higher expression levels of Fgf21 in AF patients compared with SR patients, which might be due to enhanced oxidative stress in AF patients. However, because the secretion quantity of Fgf21 from cardiomyocyte is low, it can only provide a finite-size effect. We found that administration of Fgf21 (20 nM) exerted anti-arrhythmic effects on AngII-treated mice as shown by the decrease of incidence of AF. Besides, treatment with Fgf21 remarkably improved the CV of the left atria, which may be due to suppressed atrial fibrosis. The result that APD prolonged in AngII-treated mice was consistent with previous research (10), which reflected electrical remodeling in the atria caused by AngII, while these electrical remodeling was partly recovered by Fgf21 treatment. It indicates the potential application of Fgf21 in the prevention of AF in the future.

Recent study revealed that Fgf21 decreased ROS to diminish oxidative stress by activating the expression of UCP3 and SOD2 (21). Besides, the heart from Fgf21^−/−^ mouse showed how more strongly oxidative stress responded to ISO (22). Our results were in accordance with previous studies that Fgf21 enhanced UCP3 and SOD2 levels and reduced atrial oxidative stress. NADPH oxidase (NOX2 and NOX4) have emerged as major source of ROS in AF. However, Fgf21 did not impact the expression of NOX2 and NOX4, which suggested that Fgf21 did not decrease ROS production.

Myofibril degradation is an essential aspect of structural remodeling. Research has shown that oxidative stress is involved in the process of remodeling (2). In light of Fgf21 can decrease oxidative stress, we next investigated whether Fgf21 prevents cellular myofibril degradation. The immunofluorescence results and the levels of Troponin I (cTnI) showed that Ff21 protects against tachycardia-induced myolysis. Furthermore, calpain, which contributes to the tachycardia-induced myofibril degradation, was also decreased by treating with Fgf21. Cardiac fibrosis is another important part of structural remodeling, which is characterized by fibroblast activation and altering of the extracellular matrix. A more recent study suggested that Fgf21 enables the prevention or reverse the cardiac fibrosis in a mouse model with hypertensive heart disease (7). Here, we also confirmed that Fgf21 is able to repress the phenoconversion of cardiac fibroblasts into myofibroblast. We further showed that Fgf21 decreased tachycardia-induced Tgf-β expression, which is an important activator of the myofibroblast. These results suggested that Fgf21 not only has direct protective effect on fibroblasts but also impacts cardiomyocytes to suppress fibrosis. However, the mechanisms underlying the effect of Fgf21 on fibrosis need to be further investigated.

CaMKII is now widely reported to be involved in cardiac arrhythmias (17). Ox-CaMKII can phosphorylate RyR2 serine 2814, which promotes diastolic sarcoplasmic reticulum Ca^2+^ release leading to trigger AF (17). Our results showed that Fgf21 can alleviate ox-CaMKII as well as p-RyR2 levels, which may suppress the susceptibility of AF. Other than that, Fgf21 reversed the decrease of LCC level; it suggested that Fgf21 had an effect on the electrical remodeling. It is well-known that AngII could increase ROS, enhance ox-CaMKII level, promote cardiac fibrosis, and degrade cardiac function, which favors AF. *In vivo* study, we showed Fgf21 decreased AF inducibility in AngII-treated mouse, accompanied by a reduction in the levels of ROS, ox-CaMKII, and fibrosis.

Nrf2 is recognized as the “master regulator” of the antioxidant response which regulates the expression of many genes. Several studies demonstrated Nrf2 pathway is an essential mechanism to protect cells from oxidative stress damage and Fgf21 can take effect through Nrf2 signaling pathway (23, 24). Therefore, we considered whether Fgf21 induced anti-oxidant gene expression through Nrf2. The results showed that Nrf2 silence inhibited the part of protective effect of Fgf21 on atrial remodeling. However, more studies are needed to identify the precise mechanism.

## Conclusion

In this study, we identified that Fgf21 provides protection against atrial remodeling via reducing oxidative stress and these protective effects might partly exert through Nrf2.

## Data Availability Statement

The original contributions presented in the study are included in the article/Supplementary Material, further inquiries can be directed to the corresponding authors.

## Ethics Statement

The studies involving human participants were reviewed and approved by Clinical Research Ethics Committee of the First Affiliated Hospital, Zhejiang Unoversity School of Medicine. Written informed consent for participation was not required for this study in accordance with the national legislation and the institutional requirements. The animal study was reviewed and approved by The Tab of Animal Experimental Ethical Inspection of the First Affiliated Hospital, Zhejiang University School of Medicine.

## Author Contributions

XX and LZ conceptualized and designed the study. MC, ZW, JZ, and HX completed most of the experiments. HC, XS, and LC analyzed and interpreted the patient data and performed the histological examination of the atrial tissue. MC analyzed the data and wrote the manuscript. LZ and JZ revised the manuscript critically. All the authors have read and approved the manuscript.

## Funding

This study was supported by the National Key R&D Program of China (Grant No. 2016YFC1301003), the Natural Science Foundation of China (Grant Nos. 81873484 and 81270002), and the Natural Science Foundation of Zhejiang Province, Zhejiang, China (Grant No. LQ19H070002).

## Conflict of Interest

The authors declare that the research was conducted in the absence of any commercial or financial relationships that could be construed as a potential conflict of interest.

## Publisher's Note

All claims expressed in this article are solely those of the authors and do not necessarily represent those of their affiliated organizations, or those of the publisher, the editors and the reviewers. Any product that may be evaluated in this article, or claim that may be made by its manufacturer, is not guaranteed or endorsed by the publisher.
