# Supplementary material for: Fibroblast Growth Factor 21 Protects Against Atrial Remodeling via Reducing Oxidative Stress
Source: Front Cardiovasc Med. 2021 Oct 11;8:720581. doi: 10.3389/fcvm.2021.720581 (PMC8542911; doi:10.3389/fcvm.2021.720581)

Fibroblast growth factor 21 protects against atrial remodeling via reducing oxidative stress

Miao Chen[1], Zhen Wang[1], Hongfei Xu[2], Xingang Sun[1], Heng Chen[1], Yunlong Lu[1], Lu Chen[1], Xudong Xie[1,*], Liangrong Zheng[1,*].

1 Department of Cardiology and Atrial fibrillation Center, the First Affiliated Hospital, School of Medicine, Zhejiang University, Hangzhou 310003, Zhejiang, China

2 Department of Cardiovascular Surgery, the First Affiliated Hospital, School of Medicine, Zhejiang University, Hangzhou 310003, Zhejiang, China

*Corresponding author.

Liangrong Zheng. Tel: +13805749450; fax: +13805749450. E-mail address: 1191066@zju.edu.cn.

Xudong Xie. Tel: +13857117908; fax: +13857117908. E-mail address: xiexdzju@163.com

Supplemental Table 1. Clinical characteristics of patients involved in the study

	Sex	Age	HD	AF history (months)	EF (%)	LAD (mm)
SR	M	50	AR		61	35
	F	46	MS AS AR		64	23
	M	34	AS AR MR		62	40
	M	64	AS AR		68	37
	M	52	MR		70	26
	F	73	MR		71	25
	M	52	MR		67	37
	M	48	MR		59	32
	M	65	MS MR		69	37
	M	67	AS MR		65	47
	F	36	MD		72	26
AF	M	66	MS MR	2	56	50
	F	36	MR	12	57	33
	F	63	MR AR	6	59	46
	M	47	MR MS AR	9	61	45
	F	38	MR	4	54	27
	F	69	MS MR AS AR	2	59	29
	M	39	MR AR	1	56	61
	M	42	CHD HF	2	45	40

AF atrial fibrillation, HD heart disease, EF ejection fraction, LAD left atrial dimension, SR sinus rhythm, CHD coronary heart disease, MR mitral regurgitation, MS mitral stenosis, AR aortic regurgitation, AS aortic stenosis.

Figure 1. The effects of Fgf21 on oxidative stress in primary cardiomyocytes.

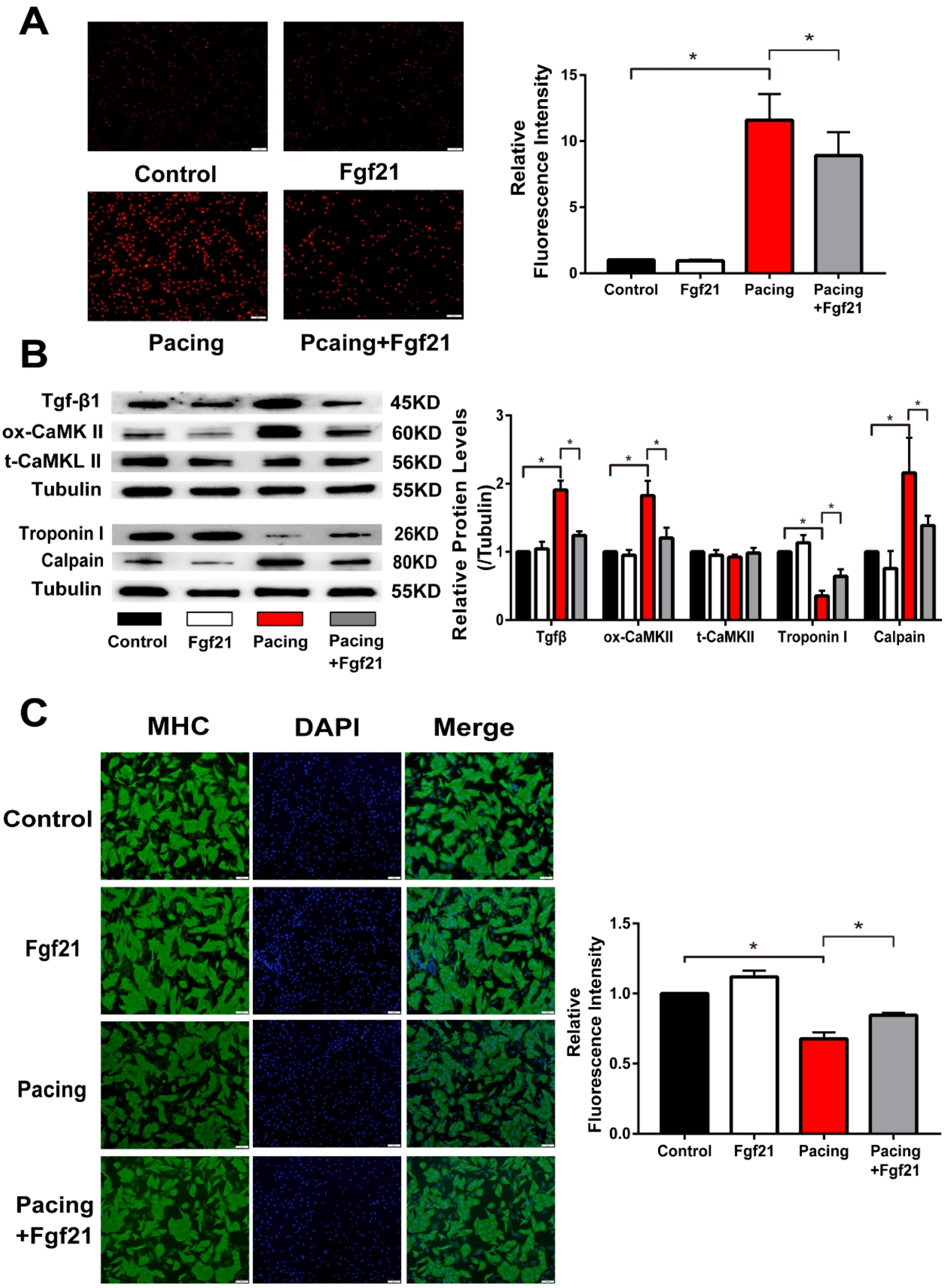

A, Representative images of ROS detected by DHE and summary of the relative fluorescence intensity of ROS in each group of primary cardiomyocytes. **B,** The expression of Tgf-β, ox-CaMKII, t-CaMKII, Troponin I, and Calpain at protein levels in each group of primary

cardiomyocytes. **C,** Immunofluorescence analysis was performed to analysis MHC, and summary of relative fluorescence intensity of MHC in each group of primary cardiomyocytes.

Figure 2. Schema shows the protective effect of Fgf21 on atrial remodeling.

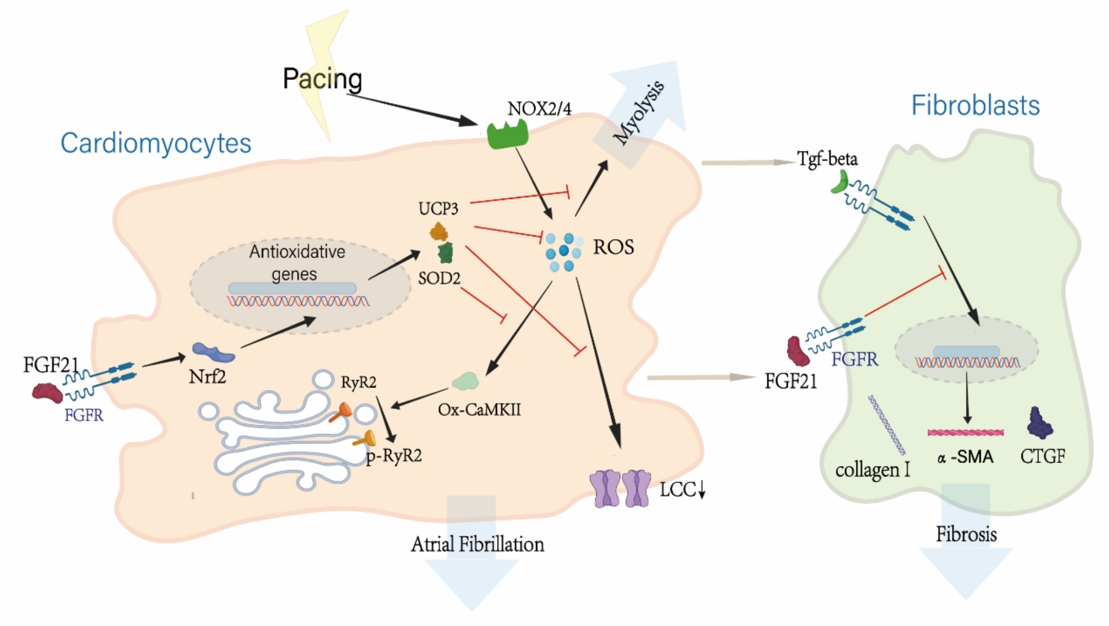

Supplement: Supplementary file 1 [file Data_Sheet_1.PDF]
